# Transnasal Transsphenoidal Approach for a Nine-Year-Old Child With Pituitary Apoplexy: A Case Report

**DOI:** 10.7759/cureus.64525

**Published:** 2024-07-14

**Authors:** Ahmed A Al-Juboori, Saif A Badran, Ali A Shahadha, Ali S Alsamok, Mustafa Ismail

**Affiliations:** 1 Department of Neurosurgery, Dr. Sa’ad AL-Witri Hospital for Neurosciences, Baghdad, IRQ; 2 Department of Surgery, Ibn Sina University of Medical and Pharmaceutical Sciences, Baghdad, IRQ; 3 Department of Surgery, College of Medicine, University of Baghdad, Baghdad, IRQ

**Keywords:** pituitary apoplexy, visual decline, mri diagnosis, hormone deficiencies, transsphenoidal, pediatric

## Abstract

Pituitary apoplexy is a rare but potentially life-threatening condition of sudden hemorrhage or infarction within the pituitary gland that results in symptoms of acute onset such as severe headache, visual impairment, and hormonal deficiencies. Though more common in adults, the same criteria for diagnostic and management dilemmas apply to pediatric cases. We present the case of a nine-year-old boy presenting with acute-onset severe headache and significant visual deterioration suggestive of pituitary apoplexy. An emergency MRI was performed, which showed a hemorrhagic sellar and suprasellar mass compressing the optic chiasm. Given the severe visual symptoms in this case, emergency surgical intervention was indicated. Decompression and gross total resection of the tumor were successfully attained using the endoscopic transnasal transsphenoidal approach by a multidisciplinary team. After the surgery, there was a significant improvement in the visual field, especially regarding the right eye's nasal hemifield, and the motor strength and consciousness remained stable. This case underscores the importance of early diagnosis and expedited surgical management in pediatric pituitary apoplexy. The transnasal transsphenoidal approach is practical for maximal decompression of the optic apparatus and reduces the risk of long-term visual deficits. In addition, it points out the need for coordinated, multidisciplinary treatment with the participation of neurosurgeons, endocrinologists, and pediatricians both for immediate and long-term consequences, including potential hormonal deficiencies. The report emphasizes the need for vigilance and prompt intervention in pediatric presentations, unlike the index case, for better outcomes and to avoid permanent morbidity.

## Introduction

Pituitary apoplexy is a rare, life-threatening condition that occurs suddenly, causing a severe headache, visual problems due to chiasmal compression, oculomotor palsy, and endocrinopathy due to hemorrhagic or non-hemorrhagic necrosis of the pituitary gland [1,2]. Although it is more frequently observed in adults, pituitary apoplexy can also present within the pediatric and adolescent populations and pose some of the most significant challenges in diagnosis and management. Picón Jaimes et al. conducted a systematic review where they analyzed eight documented international cases of pediatric pituitary apoplexy. The average age at the time of presentation was 12.8 years. The clinical manifestations included severe headache in 75% of patients, visual disturbances, nausea and vomiting in 37.5% of patients, fever in 25% of patients, and cranial nerve involvement. One patient, representing 12.5% of the total, had an altered mental status [1].

Regarding apoplexy caused by pituitary adenomas in pediatric patients, this accounts for a small fraction of intracranial neoplasms and is particularly complex in its management. The fact that early diagnosis and treatment are crucial for reducing severe effects (mainly visual disturbances) due to the disease in this very young age group was also pointed out by Zijlker et al. [2]. Clinical presentation in adolescents varies and is often with intense headaches, nausea, and visual disturbances, which warrants a high suspicion index for timely intervention.

Rovit and Fein emphasized the critical nature of pituitary apoplexy as a surgical emergency; it requires urgent intervention to prevent irreversible damage to the optic apparatus and surrounding structures [3]. Furthermore, Wang et al. in their comparative analysis wrote on the importance of early surgical management to salvage visual functions among pediatric patients with pituitary adenomas in a state of apoplexy [4]. Additionally, in recent findings, sometimes pituitary apoplexy in pediatric patients is related to underlying conditions, such as SARS-CoV-2 infections, complicating the clinical picture and mandating a thorough diagnostic approach to rule out co-infections [5].

This report outlines a case of clinical presentation, workup challenges, and management of a nine-year-old male with pituitary apoplexy which is, as far as we know, he is the youngest reported case of this condition. This study aims to illustrate the effectiveness of the transnasal transsphenoidal approach as a surgical strategy in the management of pituitary apoplexy and to underscore the significance of fast intervention and multidisciplinary management in such critical pediatric cases.

## Case presentation

A nine-year-old boy was brought to the emergency department with acute-onset severe headache and frank visual deterioration. He had lost vision in the left eye with visual acuity in the right eye with only light perception, relative afferent pupillary defect (RAPD), exodeviation, and poor convergence, and fundoscopy showed no signs of disc swelling or atrophy. His power was five on the Medical Research Council (MRC) scale for muscle strength at presentation, and his Glasgow Coma Scale (GCS) was 15. The laboratory investigations showed no hormonal disorder.

**Figure 1:**
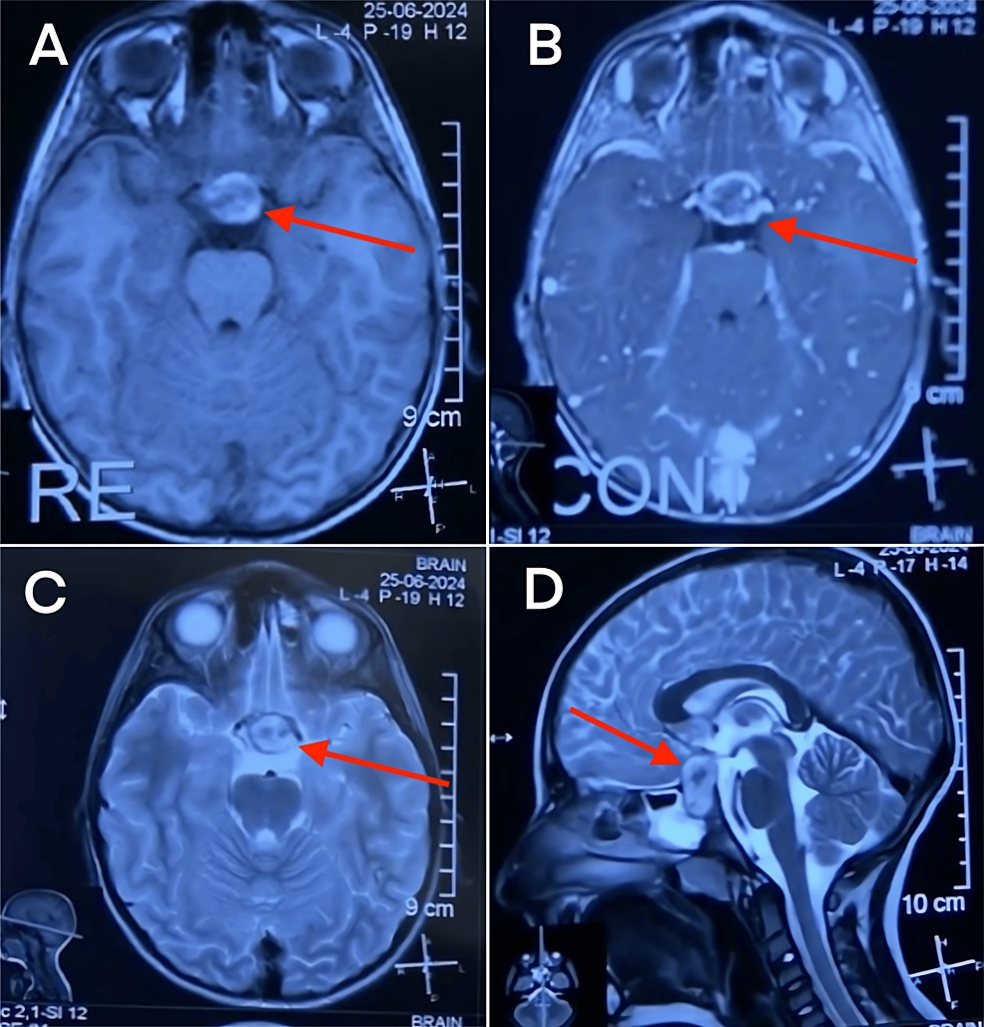
MRI images illustrating a case of pituitary apoplexy A: Axial T1-weighted MRI sequence without contrast. The image shows a mixed-intensities lesion in the pituitary gland, indicating hemorrhage within the lesion (red arrow). B: Axial T1-weighted MRI sequence with contrast. This sequence highlights the contrast enhancement around the lesion, suggesting a possible infarction within the pituitary gland (red arrow). C: Axial T2-weighted MRI sequence. The image reveals a hyperintense signal in the pituitary gland, consistent with edema or hemorrhage (red arrow). D: Sagittal T2-weighted MRI sequence. This image provides a clear view of the lesion's position within the sella turcica and its relationship to the optic chiasm and surrounding structures (red arrow).

The MRI showed a sellar and suprasellar mass lesion of about 1.5x1.5 cm in size with hemorrhagic components compressing the optic chiasma to a large degree, which was suggestive of pituitary apoplexy (Figure 1). The differential diagnosis included craniopharyngioma, Rathke cleft cyst, ruptured dermoid cyst, ruptured teratoma, paraclinoidal aneurysms, and tuberculum sellae meningiomas. He was stabilized with corticosteroids for treating inflammation and edema. Because of the severe visual symptoms, a multidisciplinary team decided upon a prompt surgical treatment. Decompression of the pituitary mass through the endoscopic transnasal transsphenoidal approach resulted in a gross total resection of the tumor (R0 residual tumor). The surgery went uneventfully.

**Figure 2:**
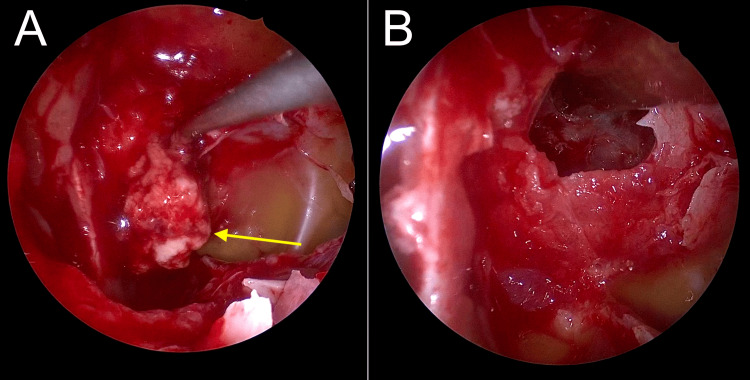
Intraoperative images of transnasal transsphenoidal endoscopic surgery A: Endoscopic view during the removal of the pituitary tumor. The yellow arrow points to the tumor tissue being resected. B: Post-resection view of the sella turcica, demonstrating the removal of the tumor (empty bed).

In the operative findings, the neurosurgeon used the "three-hand technique," and the endoscope was placed superiorly to avoid conflict between the instruments. The base of the skull was drilled cautiously with soft, un-forceful movements, and through minimum precise instruments, the mass was resected so that no critical structures were being impaired (Figure 2). One of the striking findings intraoperatively is the almost pneumatization of the sphenoid air sinuses at this age which is nine years old. The patient started to get some visual restoration in the right eye, especially in the nasal hemifield. His MRC scale was kept at 5, and his GCS was kept at 15. At the 10-day follow-up, there was significant improvement in his vision, especially in the right eye.

## Discussion

Pituitary apoplexy is a rare but critical condition that noticeably challenges outcomes. It refers to the sudden onset of pituitary gland dysfunction manifested by massive headaches and visual deterioration with hormonal deficits following hemorrhage or infarction within the pituitary gland. Although most often reported in adults, its presence in children, as presented in this case of a nine-year-old boy, contributes to the necessity of early diagnosis and management and is to be kept in mind during the management.

The patient here had characteristic symptoms of pituitary apoplexy such as a severe headache, tremendous visual decline, nausea, and vomiting. These signs and symptoms are in agreement with those reported by Zijlker et al., with an emphasis on early diagnosis and treatment to reduce mortality and long-term morbidity [2]. Neurologic examination was positive for frank visual acuity and visual field impairment as mentioned. Pituitary hormones were normal, maybe due to the short period from the onset till diagnosis, which is quite shorter than the half-life of pituitary hormones.

MRI had enormous importance in the diagnosis and detected a hemorrhagic mass in the sellar area. The imaging workup of this patient was crucial in indicating the proposed surgical management, as commented by Jankowski et al., according to whom the results of detailed neuroimaging for the assessment of the volume of hemorrhage and its impact on adjacent structures would dictate whether surgical intervention is warranted or not [6]. The transnasal transsphenoidal approach was decided, based on an urgent need to decompress the optic chiasm, and was supported by positive outcomes observed in similar cases treated surgically [1].

Indeed, the surgical intervention in this case was successful since the patient improved postoperatively; the headache and visual symptoms significantly improved. The current case is similar to a report by Wang et al., who found early surgery to be pivotal for salvaging visual function and avoiding permanent deficits in pediatric patients with pituitary apoplexy [4]. We could substantially reduce the size of the pituitary mass and relieve the compression over the optic apparatus in the patient using a transnasal transsphenoidal approach.

Long-term hormone deficiencies should be managed in cases of pituitary apoplexy such as those that had been underscored by Yang et al. in the continued endocrine therapy for the treatment of deficiencies caused by pituitary damage [7]. On the other hand, Pinto et al. have also underscored that pituitary apoplexy may lead to severe endocrine dysfunctions that would necessitate lifelong hormone replacement [8].

A thorough review of the pneumatization of the sphenoid sinus in children indicates that it generally commences at approximately two months of age and is typically fully developed by the time the child reaches 12 to 14 years old. Pneumatization is observed to a large extent in most youngsters between the ages of three and six [9]. An MRI of our nine-year-old male patient with pituitary apoplexy revealed nearly total pneumatization of the sphenoid sinus. The discovery is highly unusual for someone of his age, as complete air cavity formation typically takes place between the ages of 12 and 14 years. This highlights the advanced growth of the sphenoid sinus in this patient, which enabled the successful adoption of the transnasal transsphenoidal approach in his treatment.

This case illustrates that the management of pituitary apoplexy in children is multidisciplinary, including neurosurgeons, otolaryngologists, endocrinologists, and pediatricians. Good care requires an early surgical operation, cautious perioperative care, and close long-term follow-up to manage and monitor hormonal deficiencies. The following case report highlights how practical a comprehensive approach can be [10].

In summary, the case shows that apoplexy of the pituitary gland is a life-threatening condition in the pediatric age group that needs urgent multidisciplinary treatment. Successful decompression via the transnasal transsphenoidal approach with significant improvement of visual and hormonal symptoms was accomplished. Early surgical intervention, combined with detailed management of postoperative endocrinopathy if existing, proved crucial to the outcome. This case serves as a reminder that suspicion must be kept high, with quick thinking in a similar presentation to prevent long-term complications.

## Conclusions

Early, accurate diagnosis of this nine-year-old boy with apoplexy of the pituitary was essential for timely multidisciplinary intervention in a pediatric setting. Visual functions were restored, and the hormonal state normalized by decompression via a transnasal, transsphenoidal approach. In this case, clinical pearls included that very severe visual loss can be avoided with rapid surgical decompression, and postoperative endocrine management needs to be long-term to follow up on potential postoperative hormonal deficiencies. This case points to the importance of a high index of suspicion and quick coordination of action in preventing further long-term complications in similar pediatric presentations.
